# Seeking Health Information and Support Online: Does It Differ as a Function of Engagement in Risky Health Behaviors? Evidence From the Health Information National Trends Survey

**DOI:** 10.2196/jmir.3368

**Published:** 2014-11-06

**Authors:** Lion Shahab, Jamie Brown, Benjamin Gardner, Samuel George Smith

**Affiliations:** ^1^Department of Epidemiology and Public HealthUniversity College LondonLondonUnited Kingdom; ^2^Department of Clinical, Educational and Health PsychologyUniversity College LondonLondonUnited Kingdom; ^3^Department of General Internal Medicine and GeriatricsNorthwestern UniversityChicago, ILUnited States

**Keywords:** health-risk behavior, online support, Internet-based intervention, information seeking, health information and national trends survey (HINTS)

## Abstract

**Background:**

The Internet is an important tool to deliver health behavior interventions, yet little is known about Internet access and use of health-related information, or support, by the intended intervention recipients.

**Objective:**

Our aim was to evaluate whether health-related Internet use differed as a function of common health-risk behaviors (excessive alcohol consumption, smoking, low fruit/vegetable intake, inactive/sedentary lifestyle, unprotected sun exposure, or obesity).

**Methods:**

Sociodemographic, health behavior characteristics, and information on Internet access and use were assessed in the nationally representative US Health Information National Trends Survey (HINTS) 4. Data from 3911 participants collated in 2011/12 were included.

**Results:**

Of the 78.2% (95% CI 76.1-80.1) of participants who had ever accessed the Internet, approximately three-quarters (78.2%, 95% CI 75.4-80.7) had obtained health-related information online last year. About half had used the Internet as the first source of health-related information (47.8%, 95% CI 44.8-50.7) or to access behavioral support (56.9%, 95% CI 53.7-60.0) in the last year. Adjusting for sociodemographic determinants of going online (being younger, white, female, with at least college education) revealed few differences in Internet access and use between health-risk behaviors. Participants with inadequate sun protection were less likely to access the Internet (OR 0.59, 95% CI 0.04-0.88) and those with low fruit/vegetable intake were less likely to have gone online to obtain health-related information last year (OR 0.60, 95% CI 0.45-0.80). Smokers in particular were likely to use the Internet to obtain behavioral support (OR 1.90, 95% CI 1.35-2.68).

**Conclusions:**

Internet access and use to obtain health-related information and support is widespread and mostly independent of engagement in various health-risk behaviors. However, those with low fruit/vegetable intake or inadequate sun-protective behaviors may be more difficult to reach with Internet-based interventions. In addition, when developing online health promotions, relevant sociodemographic determinants of Internet use need to be targeted to maximize their impact.

## Introduction

Over the last decade, global access to the Internet has dramatically increased such that over 80% of the US population now uses the Internet [1]. A similar proportion access the Internet in other developed countries such as in the United Kingdom [2], and worldwide one in three people are now connected online [3]. This has been accompanied by a proliferation of online sources of health-related information and support [4]. The use of the Internet to promote health and deliver interventions can engage those reluctant to use face-to-face support by providing an anonymous environment that ensures confidentiality and reduced stigma. Internet-based interventions offer a convenient means of helping those who would otherwise struggle to access face-to-face support due to mobility or geographical barriers, while offering a cheaper and more scalable alternative to offline health interventions [5].

Health interventions try to modify health-risk behaviors, which can be defined as actions that cause preventable morbidity and mortality. Tobacco smoking and overeating alone contribute to 8 million avoidable global deaths every year [6], and over a third of cancer cases are attributable to health-risk behaviors [7]. The proportion of US adults meeting daily recommendations for fruit and vegetable intake [8] and physical activity is inadequate [9], and tobacco smoking prevalence remains above 18% [10]. Despite decades of large-scale health promotion campaigns and interventions, the number of deaths attributable to health-risk behaviors is projected to increase even further [11]. Therefore, there is a continued need to tackle these behaviors.

It is encouraging that Internet-based interventions, as a novel way to engage those who persist with health-risk behaviors, have been shown to have a small but clinically significant effect on promoting health behavior change [12]. For instance, there is evidence from controlled trials that interactive, online interventions for tobacco use that personalize information and provide tailored feedback can increase 6-month abstinence rates by 17% [13]. Similarly, online interventions that provide personalized feedback and normative information have been shown to reduce weekly alcohol consumption by around 0.5 standard (10 mg) units [14]. Web-based exercise interventions that involve goal setting and online coaching can result in small but positive increases in physical activity [15]. Online interventions for obesity that provide behavior therapy and e-counseling have yielded weight loss of up to 7 kg over 6 months to 1 year [16]. Internet-based interventions also have the potential to address social health inequalities within and between countries [17] that are attributable to health-risk behaviors [18]. Interest in online support appears to be equally spread across the social spectrum [19], and there is evidence of a decreasing digital divide [20]. The universal ease of accessing Internet-based interventions is therefore a potential asset in the quest towards decreasing inequality and improving the health outcomes of the poorest in society.

However, despite the recent proliferation of eHealth, relatively little is known about the actual reach of Internet interventions [21], and there remains a need to increase exposure to health life-style interventions delivered online [22]. For instance, it is currently unclear whether the Internet, and Internet-based health-related information and support, are accessed to the same degree and in a similar manner by people who do or do not engage in health-risk behaviors. Given that overall intervention impact is determined by both efficacy and reach, this information is important for evaluating the potential of interventions to improve health behaviors.

It is possible that the Internet and Internet-based support are accessed either more or less frequently by those who are the intended target. If the former is the case, then this further adds to the potential of the Internet as a preferred medium to deliver health interventions. Yet, if the latter is the case, then Internet-based interventions may not be as beneficial as assumed and may have suboptimal real-world effectiveness at population level despite proven efficacy in clinical trials. This would require that dissemination channels for Internet-based interventions be changed, for instance, by making intended users aware of such interventions through their health care providers or by using targeted marketing. Additionally, knowing more about what kind of person does or does not engage with eHealth can inform intervention design, for example, in terms of providing adequate or enhanced functionality and effective tailoring based on user characteristics to encourage those who are currently not making the most of the Internet to use this resource to improve their health [23].

Data are therefore needed on access to and reach of Internet-based interventions as well as sociodemographic determinants of use to aid development and optimization of online material. As North America has one of the highest rates of penetration of Internet access [3] and most mature online markets for eHealth [4], we sought to provide these data in a US sample. We addressed the following research questions:

1. What is the prevalence of general Internet use, and does this differ as a function of sociodemographic characteristics and engagement in specific health-risk behaviors?

2. What is the prevalence of Internet use to access health-related information and support online, and does this differ as a function of sociodemographic characteristics and engagement in specific health-risk behaviors?

## Methods

### Study Population and Design

**Figure 1:**
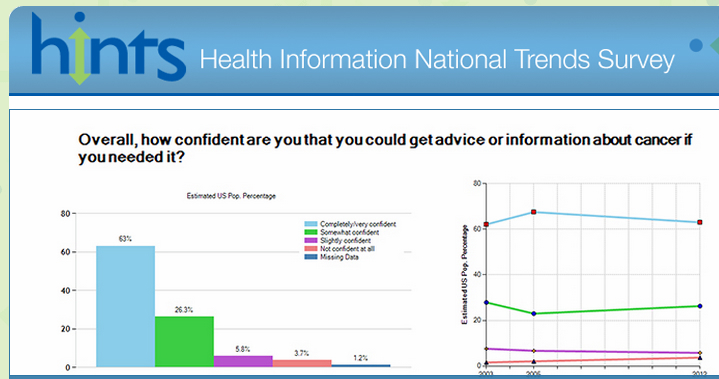
HINTS screenshot.

Data come from Health Information National Trends Survey (HINTS) 4 (Cycle 1), a national probability survey of adults aged 18 or older in the civilian non-institutionalized population of the United States that assesses usage and trends in health information access and understanding (Figure 1). This study uses data from the fourth data collection wave, carried out between October 2011 and February 2012 by the National Cancer Institute. A full description of HINTS methodology is available elsewhere [24,25]. Briefly, the latest iteration used a two-stage stratified sample of addresses present on the Marketing Systems Group database to which questionnaires were mailed for self-administration (in both English and Spanish). This was followed by a reminder card and an additional three mailings of the questionnaire depending on non-response. The sampling frame of addresses was divided into high and low ethnic/racial minority strata, with high minority areas oversampled to provide more exact estimates for minority populations. For each selected household, participants were identified by randomly allocating household to one of two selection methods. In the next-birthday method, the adult in the household whose birthday is soonest completes the survey, and in the all-adult method, any adult in the household can complete the survey. Response rates for the two methods were 37.9% and 35.3% respectively, yielding an overall response rate of 36.7%.

### Measures

#### Sociodemographics

Age, employment status (employed; yes/no), marital status (married; yes/no), ethnicity (white; yes/no), and educational attainment (college education or above; yes/no) were recorded. General health was assessed with an established single item asking participants to rate their health as “excellent”, “very good”, “good”, “fair”, or “poor” [26]. The latter two and former three categories were respectively combined to create a binary health status variable (poor health; yes/no).

#### Health-Risk Behaviors

Alcohol consumption was determined by asking on how many days per week during the last 30 days participants had at least one drink of an alcoholic beverage (defined as a standard measure of alcohol in beer, wine, wine cooler, cocktail, or other liquor). Participants were also asked how many drinks they consumed on the days they did drink. US guidelines for alcohol consumption state that moderate alcohol consumption constitutes an average of one drink per day for women and two drinks per day for men [27]. This information was used to calculate a binary variable reflecting alcohol consumption above these levels (excessive alcohol use; yes/no).

Cigarette smoking was assessed by asking participants whether they had smoked at least 100 cigarettes in their lifetime, and if so, whether they smoked every day, some days, or not at all nowadays. This information was used to calculate a binary variable reflecting any current (daily or non-daily) cigarette use (current smoking; yes/no).

Diet was assessed by asking participants how many cups of fruit (including 100% pure fruit juice) or vegetables (including 100% pure vegetable juice) they consumed each day. Examples of what a cup means (eg, one large banana, 12 baby carrots) were provided. Based on standard guidelines recommending at least 5 servings (roughly equivalent to 2.5 cups) of fruit and vegetables per day [28], a binary variable reflecting restricted dietary intake was computed (low fruit/vegetable intake; yes/no).

Physical activity was determined by asking on how many days a week participants engaged in bouts of exercise of at least moderate intensity, and how long a typical bout lasted. In addition participants were asked how many hours per day on average they sat and watched TV or movies, surfed the Web, or played computer games (excluding active gaming). US guidelines recommend at least 30 minutes of moderate physical activity on 5 days a week [29] and accumulating evidence links excessively inactive leisure time behavior to increased mortality [30]. This information was therefore combined into a binary variable (inactive/sedentary lifestyle; yes/no) to identify those with both inadequate physical activity (no moderate activity/exercise) and high “screen time” (≥4 hours per day).

Sun-protective behavior was assessed by asking participants how many times they had used a tanning bed or booth in the last year, as well as whether and how often they use sunscreen when outside for more than one hour on a sunny day (always, often, sometimes, rarely, never; do not go out on sunny days). Following sun-safe guidelines that recommend minimizing exposure to ultraviolet radiation [31] by avoiding tanning beds and always wearing sunscreen, responses were used to compute a binary variable of sun-safe behavior (unprotected sun exposure; yes/no).

Participants also self-reported anthropometric measures that were converted into Body Mass Index (BMI in kg/m^2^) and used to compute obesity (BMI≥30; yes/no).

Internet use and access to health information online were measured by the following:

- Ever use of Internet: Access to the Internet was established by asking participants whether they ever went online to access the Internet or to send and receive emails.
- Internet use for health-related information last year: Access to online health information was determined by asking whether participants had used the Internet in the last year to look for health or medical information for themselves.
- Internet first source for health-related information: Participants were asked to pick one item from a list to indicate where they would first go if they had a strong need to get information about health or medical topics. The list comprised family, friends/co-workers, doctors/health care professionals, books, brochures, libraries, specialized organizations, magazines/newspapers, complementary/alternative practitioner, telephone helpline, or the Internet. This list was used to create a variable to denote use of the Internet as a first port of call for health-related information.
- Internet use for behavioral support last year: Participants were prompted to indicate various specific uses of the Internet over the last year (eg, to buy medicine or vitamins online, to look for health care providers, to write an online diary or blog on health topics). This list of uses was used to create a variable denoting use of the Internet for behavioral support (use of websites to help with diet, weight, smoking cessation, or physical activity; participation in online support groups for people with similar health or medical issues; downloading of health-related information to a mobile device or visiting “a social networking” site to read and share about medial topics).

### Analysis

Out of a total of 3959 participants, only those who provided information on Internet use and at least one health-risk behavior (3911/3959, 98.79%) were included in the analytic sample. In univariable analysis, differences in categorical and continuous variables between those who did and did not access the Internet, or between those who did and did not use online resources for health-related information and support, were compared with chi-square and *t* test, respectively. In multivariable analysis that controlled for sociodemographic characteristics, the association between health behaviors and Internet access and use was determined with logistic and linear regressions. Sampling weights based on the Horvitz-Thompson estimator to account for sample design and non-response (jackknife with 50 replicates) were applied to all analyses to calculate accurate standard errors of estimates [32]. This was implemented using the svyset command in STATA version 12 to declare the appropriate survey design.

## Results

### Overview

As shown in Table 1, poor sun-protective behavior was by far the most prevalent of the health-risk behaviors; nearly nine out of ten participants did not follow sun-safe guidelines (87.7%, 95% CI 86.4-88.9). This was followed by low fruit/vegetable intake (56.0%, 95% CI 53.6-58.3), obesity (28.9%, 95% CI 26.9-31.0), current smoking (17.8%, 95% CI 15.8-20.0), excessive alcohol consumption (15.1%, 95% CI 12.8-17.7), and having a sedentary life-style (14.5%, 95% CI 12.5-16.9).

**Table 1:** Univariable associations of health-related Internet use with sociodemographic, health characteristics, and health-risk behavior.

		Total^a^ (N=3911)	Ever use of Internet		Internet use for health-related information last year^b^		Internet first source for health-related information^b^		Internet use for behavioral support last year^b^	
			Yes (n=2886)	No (n=1025)	Yes (n=2222)	No (n=650)	Yes (n=1318)	No (n=1459)	Yes (n=1321)	No (n=1236)
**Sociodemographic & health characteristics**										
	Age in years, mean (SD)	46.35 (18.01)	42.88 (15.57)	58.91 (21.00)^c^	42.48 (15.69)	44.06 (17.04)	42.30 (14.82)	43.71 (16.94)	39.78 (13.99)	47.59 (17.70)^c^
	Male, % (n)	48.50 (1576)	47.87 (1130)	50.76 (446)	45.38 (841)	57.14 (286)^c^	50.00 (524)	45.95 (568)	45.02 (449)	51.78 (550)^c^
	White, % (n)	80.54 (2819)	82.05 (2183)	74.87 (636)^c^	82.41 (1689)	82.00 (486)	81.39 (1023)	83.09 (1089)	79.11 (978)	84.83 (964)
	Married, % (n)	51.23 (2015)	52.75 (1604)	45.66 (411)^c^	52.79 (1241)	53.54 (359)	52.85 (732)	54.30 (816)	49.96 (697)	58.53 (732)^c^
	Employed, % (n)	55.90 (2036)	61.28 (1756)	36.26 (280)^c^	61.93 (1396)	59.82 (354)	63.08 (854)	59.53 (837)	62.83 (878)	60.74 (678)
	College education, % (n)	64.10 (2672)	73.88 (2311)	28.08 (361)^c^	77.47 (1835)	61.83 (467)^c^	78.13 (1111)	71.79 (1118)^c^	77.51 (1103)	72.73 (972)
	Poor health, % (n)	15.04 (623)	12.36 (343)	25.00 (280)	12.97 (268)	9.97 (71)	14.19 (156)	10.82 (174)	13.92 (161)	9.93 (132)
	BMI, mean (SD)	27.66 (6.53)	27.51 (6.32)	28.25 (7.27)	27.50 (6.35)	27.59 (6.99)	27.52 (6.36)	27.55 (6.73)	27.82 (6.77)	27.07 (6.39)
**Health-risk behavior, % (n)**										
	Excessive alcohol consumption	15.06 (484)	15.47 (368)	13.57 (116)	15.52 (286)	15.34 (80)	14.84 (178)	15.66 (181)	15.89 (190)	15.87 (141)
	Current smoking	17.78 (615)	16.15 (419)	23.71 (196)^c^	15.34 (316)	19.34 (101)	16.65 (199)	15.32 (206)	19.84 (240)	11.70 (130)^c^
	Low fruit/ vegetable intake	55.98 (2066)	54.85 (1492)	60.06 (574)	52.54 (1115)	63.72 (369)^c^	58.20 (706)	52.20 (732)^c^	54.91 (676)	54.67 (640)
	Inactive/ sedentary lifestyle	14.54 (545)	11.74 (324)	25.25 (221)^c^	10.93 (229)	14.70 (94)	13.01 (154)	11.09 (158)	12.10 (137)	11.91 (151)
	Unprotected sun exposure	87.69 (3309)	86.97 (2414)	90.29 (895)^c^	86.60 (1853)	88.33 (549)	86.97 (1119)	86.86 (1208)	87.48 (1101)	85.67 (1035)
	Obese	28.88 (1122)	27.69 (819)	33.40 (303)^c^	27.94 (638)	27.07 (177)	26.51 (356)	28.76 (434)	28.64 (417)	24.49 (299)

^a^All counts in table are unweighted.

^b^Restricted to those who have ever used the Internet.

^c^ *P*<.05.

### Prevalence of General Internet Use and Differences as a Function of Sociodemographic Characteristics and Engagement in Risky Health Behaviors

General Internet use was common as nearly four out of five participants indicated that they had ever used it (78.2%, 95% CI 76.1-80.1). Univariable analysis showed that participants who engaged in any health-risk behaviors (with the exception of excessive alcohol consumption and low fruit/vegetable intake) were significantly less likely to have ever used the Internet (see Table 1).

However, after controlling for sociodemographic and other characteristics in multivariable analysis, only participants with unprotected sun exposure remained less likely to have ever used the Internet (Table 2). Younger age, being female, married, of white ethnicity, and having a college education were all independently associated with ever using the Internet (Table 2).

**Table 2:** Multivariable associations of health-related Internet use with sociodemographic, health characteristics, and health-risk behavior.

		Ever use of Internet	Internet use for health-related information last year^c^	Internet first source for health-related information^c^	Internet use for behavioral support last year^c^
**Sociodemographic & health characteristics** ^a^ **, OR (95% CI)**					
	Age	0.94 (0.92-0.95)^d^	0.99 (0.98-1.00)	1.00 (0.99-1.00)	0.97 (0.96-0.98)^d^
	Male	0.65 (0.47-0.89)^d^	0.64 (0.45-0.90)^d^	1.11 (0.56-1.44)	0.69 (0.50-0.93)^d^
	White	2.15 (1.44-3.20)^d^	0.92 (0.57-1.49)	0.90 (0.56-1.47)	0.71 (0.46-1.10)
	Married	1.69 (1.25-2.28)^d^	1.06 (0.78-1.44)	0.95 (0.77-1.17)	0.99 (0.74-1.34)
	Employed	1.40 (0.89-2.21)	1.11 (0.77-1.60)	1.19 (0.90-1.56)	1.00 (0.69-1.45)
	College education	7.40 (5.47-10.0)^d^	2.17 (1.40-3.36)^d^	1.42 (0.97-2.08)	1.41 (1.01-1.98)^d^
	Poor health	0.79 (0.51-1.22)	1.77 (1.03-3.05)^d^	1.40 (0.83-2.37)	1.43 (0.93-2.21)
	BMI	0.99 (0.95-1.02)	1.00 (0.97-1.03)	1.00 (0.98-1.02)	1.03 (1.00-1.05)^d^
**Health-risk behavior** ^b^ **, OR (95% CI)**					
	Excessive alcohol use	0.61 (0.34-1.08)	0.86 (0.54-1.36)	0.83 (0.53-1.31)	0.86 (0.59-1.24)
	Current smoking	0.62 (0.38-1.02)	0.78 (0.47-1.29)	0.97 (0.67-1.39)	1.90 (1.35-2.68)^d^
	Low fruit/vegetable intake	1.03 (0.70-1.52)	0.60 (0.45-0.80)^d^	1.32 (1.04-1.68)^d^	0.97 (0.69-1.36)
	Inactive/ sedentary lifestyle	0.80 (0.36-1.75)	0.62 (0.37-1.05)	1.22 (0.80-1.87)	0.94 (0.53-1.68)
	Unprotected sun exposure	0.59 (0.40-0.88)^d^	0.94 (0.66-1.35)	0.97 (0.75-1.26)	1.01 (0.67-1.53)
	Obese	0.91 (0.60-1.38)	1.03 (0.72-1.49)	0.91 (0.67-1.24)	1.32 (1.00-1.75)

^a^Estimates from model including all sociodemographic & health characteristics but no health-risk behaviors. ^b^Estimates in separate models for each health-risk behavior, including sociodemographic & health characteristics covariates (BMI omitted from models with “Obese” as health-risk behavior).

^c^Restricted to those who have ever used the Internet.

^d^ *P*<.05.

### Prevalence of Internet Use to Access Health-Related Information and Support Online and Differences as a Function of Sociodemographic Characteristics and Engagement in Risky Health Behaviors

Among those who had ever accessed the Internet, over three-quarters of participants (78.2%, 95% CI 75.4-80.7) had used it to obtain health-related information during the last year. Participants with low fruit/vegetable consumption were less likely to have sought health-related information online in the last year both in univariable analysis (Table 1), and after controlling for sociodemographic and other confounders in multivariable analysis (Table 2). Being female, in poor health, and having a college education were independently associated with use of the Internet to access health-related information in the last year (Table 2).

Nearly half of Internet users reported that they would look online first whenever they urgently required health-related information (47.8%, 95% CI 44.8-50.7). A higher proportion of those with, rather than without, low fruit and vegetable intake said they would use the Internet as a first source for information on health and medical topics. This was the case both in univariable analysis (Table 1) and when adjusting for potential confounders in multivariable analysis (Table 2).

Over half of all those who had ever been online also reported using the Internet to access some sort of health-related behavioral support in the last year (56.9%, 95% CI 53.7-60.0). Both univariable (Table 1) and multivariable (Table 2) analysis showed that smokers were nearly twice as likely as non-smokers to have used the Internet to obtain behavioral support during last year. Presumably this was primarily due to getting support for stopping smoking as this difference disappeared when information seeking for quitting smoking was excluded from the definition of behavioral support (OR 1.10, 95% CI 0.71-1.69). Participants who were younger, female, college educated, and with higher BMI were also more likely to have accessed behavioral support online during the last year (Table 2).

## Discussion

### Principal Findings

Our findings provide up-to-date information on Internet access in the United States and demonstrate its widespread use to obtain health-related and medical information and support. In agreement with other national data [3,33], we find that over three-quarters of adults in the United States have ever gone online. Of these, the same proportion has used the Internet to look for health or medical information in the last year and nearly half to obtain behavioral support. This study is the first to show that there are few differences in Internet access and use for health-related support and information between people who do or do not engage in specific health-risk behaviors. This provides empirical evidence that Internet-based interventions to change health-risk behaviors generally reach those who are the intended target of health promotion and lends further credence to the potential of the Internet as a platform for improving public health [21].

Nonetheless, the findings also suggest that the Internet may not be equally effective for addressing all types of health-risk behaviors. In particular, the Internet may be less effective for promoting sun-protective behaviors and related awareness campaigns as Internet access is lower in the at-risk population, even after taking sociodemographic confounders into account. The reasons for this are unclear. It may in part reflect lower Internet penetration of rural areas where poor sun-protective behavior can be more prevalent [34,35], though the extent to which an area was urban or rural was not directly assessed in the current study. Among those with access to the Internet, participants with a diet low in fruit and vegetables were more likely to report using the Internet as the first source for health-related information but were less likely to have used the Internet to obtain health-related information in the last year. This finding is in agreement with work from the United Kingdom, which suggests that daily recommended intake of fruit and vegetables is associated with consistent Internet use after controlling for known confounders [36]. Our results also indicate that the Internet may be particularly effective for providing behavioral support for smoking cessation as current smokers were nearly twice as likely to seek support online, primarily for help with stopping smoking. However, even then, few use intensive online support to aid quit attempts [19].

There were also sociodemographic correlates of Internet use that were mostly independent of health-related behaviors. Access to the Internet and gaining health-related information and support online was associated with being younger, female, having at least college level education and less so with white ethnicity and being married. Importantly, the observed associations of health-risk behaviors with reduced access to the Internet were attenuated but not eradicated when controlling for sociodemographic determinants. Although this suggests that the Internet may be a good medium to deliver health promotion messages and interventions to those with health-risk behaviors, it also indicates a need to be aware that older, male, non-white, and less educated people could be less likely to benefit from the availability of online health-related support. Indeed, many of the characteristics that were associated with limited access or use of the Internet to obtain health-related and medical information in this study such as unemployment, worse education, and being single are also linked with detrimental health behaviors (eg, [37,38]).

Our results have a number of implications. The Internet appears to have sufficient reach to engage people who display various risky health behaviors and, given its other advantages, is therefore a good medium to deliver online interventions to address excessive alcohol use, overeating, and physical inactivity. Based on our findings, smoking cessation interventions in particular may benefit from being delivered online. However, as access to the Internet and its use for obtaining health-related information is more limited among people with inadequate sun protection and with low fruit and vegetable intake, Internet-based interventions to change these behaviors may be less effective and require additional promotion. For instance, it may be important to supplement such interventions with print material and tailored advertising in health care outlets to reach the target population. Moreover, even though access to the Internet has grown exponentially over the last 15 years, this access is not equal across all population characteristics [39], and our results highlight the need to be aware of sociodemographic determinants of Internet use for health information. In order to avoid increasing health inequalities and decreasing effectiveness, online material will need to be tailored to characteristics such as male gender, older age, and lower educational attainment to engage these users. This can be done successfully, for instance, in the area of online support for smoking cessation [40,41].

### Limitations

The study has a number of limitations inherent to most surveys. Findings rely on self-reported data, and this may have introduced biases due to systematic misreporting or forgetting. For instance, participants may underreport their alcohol [42] and tobacco consumption [43] due to social desirability concerns. Moreover, given the cross-sectional nature of the study, no causal interpretations can be made, and we cannot exclude the possibility that unmeasured confounding factors explain some of the observed associations. For example, particular trait characteristics that influence health-risk behaviors (such as greater impulsivity, which is associated with alcohol use and smoking [44]) may also determine use of the Internet to access health-related information. Notwithstanding these limitations, given the anonymous nature of data collection, misreporting is unlikely to have made a substantial contribution to results, and common confounders in the analysis were controlled. Strengths of the study include its representativeness of the US population and its large sample size. However, findings will need to be replicated in longitudinal analyses and other countries to confirm and clarify the reported associations.

### Conclusions

Overall, our results suggests that the Internet has a wide reach and should be an effective tool to provide support and information for improving most health-risk behaviors but that sociodemographic characteristics of users need to be taken into consideration when developing online health promotion material.

## Abbreviations

BMI: Body Mass Index
HINTS: Health Information National Trends Survey
